# Differentiated molecular defense strategies and survival trade-offs in the marine ciliate *Euplotes vannus* exposed to structurally homologous tetracyclines

**DOI:** 10.3389/fmicb.2026.1847705

**Published:** 2026-07-03

**Authors:** Jialu Yu

**Affiliations:** School of Marine Sciences, Ningbo University, Ningbo, China

**Keywords:** ciliates, comparative transcriptomics, defense trade-off mechanisms, marine ecotoxicology, tetracyclines

## Abstract

Antibiotic contamination, particularly by tetracycline hydrochlorides, poses a severe threat to marine benthic ecosystems. This study investigates the differentiated toxicological effects and molecular defense mechanisms of the marine ciliate *Euplotes vannus* when exposed to three structurally similar tetracyclines: chlortetracycline hydrochloride (CTC), doxycycline hydrochloride (DOX), and tetracycline hydrochloride (TCH). Through acute (6 h) and chronic (144 h) exposure assessments, we integrated population dynamics, biochemical enzyme assays, and comparative transcriptomics. The 24-h EC₅₀ values established a toxicity gradient of CTC (4.858 mg/L) > TCH (8.336 mg/L) > DOX (9.023 mg/L). Biochemical assays revealed severe lipid peroxidation across all treatments alongside distinct antioxidant enzyme activation patterns: CTC significantly induced catalase and peroxidase, whereas DOX primarily elevated superoxide dismutase activity. Transcriptomic analysis elucidated compound-specific molecular responses and survival strategies for each antibiotic. CTC exposure triggered global metabolic reprogramming, accelerating lipid and branched-chain amino acid degradation to fuel cytochrome P450-mediated detoxification and transmembrane efflux. Conversely, DOX induced profound endoplasmic reticulum stress and disrupted protein folding, necessitating the upregulation of molecular chaperones and energy systems. TCH exposure resulted in translational arrest and the global downregulation of energy metabolism, leaving *E. vannus* with a diminished compensatory antioxidant response despite sustaining the most severe lipid damage. These findings demonstrate that *E. vannus* does not deploy a uniform defense against tetracycline hydrochlorides; instead, it executes complex, compound-specific adaptive strategies involving energy reallocation, macromolecular quality control, and targeted detoxification. This study provides critical insights into the comparative ecotoxicology of marine protozoa.

## 1 Introduction

The widespread clinical and agricultural use of antibiotics causes persistent contamination of marine environments (Amangelsin et al., **2023**; Lajqi Berisha et al., **2024**). Tetracyclines (TCs) are of particular concern among these pollutants. Specific variants include chlortetracycline hydrochloride (CTC), doxycycline hydrochloride (DOX), and tetracycline hydrochloride (TCH). These compounds exhibit prolonged half-lives. Consequently, they readily accumulate in benthic sediments and interstitial waters (Amangelsin et al., **2023**; Sha et al., **2025**; Wanjeri et al., **2025**). This localized accumulation creates chronic, severe chemical stress for non-target benthic organisms. These organisms form the vital base of the marine food web. Therefore, such prolonged exposure poses unpredictable long-term ecotoxicological risks (Gomes, **2024**; Roose-Amsaleg et al., **2013**).

Protozoa, such as ciliates, act as central drivers of the microbial loop within marine food webs. They serve as critical links. They transmit biogeochemical energy to higher trophic levels (Azam et al., **1983**; Fenchel, **2008**). *Euplotes vannus* is a widely distributed benthic ciliate. It serves as an ideal ecotoxicological model organism. This species resides continuously at the sediment–water interface. Consequently, it experiences direct exposure to high pollutant loads without environmental buffering (Gong et al., **2005**). Furthermore, *E. vannus* lacks a protective cell wall or chitinous exoskeleton. Its highly permeable cell membrane makes it exceptionally vulnerable to dissolved toxicants (Lynn, **2008**; Sauvant et al., **1999**). This highly evolved unicellular eukaryote features complex organelles and metabolic networks. Therefore, it provides a unique multi-scale indicator capability. Researchers can observe macroscopic population-level dynamics alongside microscopic transcriptional reprogramming in response to stress (Tian et al., **2025**). Globally, tetracycline concentrations in surface waters typically range from 3 to 500 ng/L (Kovalakova et al., **2020**). However, Asian surface waters exhibit higher contamination. Concentrations there primarily fall between 2 and 1,000 ng/L. In aquaculture, therapeutic tetracycline baths often reach concentrations of 12.5 mg/L to treat or prevent diseases in aquatic animals (Jiang, **2000**).

Extensive empirical investigations have examined the toxic effects of persistent pollutants. However, current ecotoxicological research on marine protozoa largely relies on macroscopic phenotypic observations or single-endpoint assays. Acute lethality (EC₅₀) determinations are common examples (Fent et al., **2006**). These approaches facilitate rapid toxicity screening. Yet, they lack the mechanistic depth required to elucidate cellular survival strategies under stress (Zhang et al., **2018**). Under sublethal antibiotic stress, unicellular organisms must execute precise resource allocations within a narrow viability window. They urgently divert energy away from routine proliferation. Instead, they fund detoxification, receptor repair, and antioxidant defenses (Li et al., **2024**; Sokolova, **2013**). Broad-spectrum antibiotics do not differentiate between pathogenic bacteria and beneficial microflora (Patangia et al., **2022**). In aquatic ecosystems, this pollution inhibits crucial non-target microorganisms. It subsequently disrupts vital nutrient cycling, such as carbon and nitrogen fixation, altering the overall ecological balance (Yang et al., **2021**). Certain antibiotics, including tetracyclines and aminoglycosides, can inhibit mitochondrial protein synthesis. This inhibition causes cellular toxicity, oxidative stress, and tissue damage in non-target hosts (Chopra and Roberts, **2001**; Polianciuc et al., **2020**). Despite this, systematic molecular evidence remains scarce. The precise mechanisms by which ciliates orchestrate these defense networks are poorly understood. This is especially true when they encounter structurally homologous antibiotics with distinct functional side chains.

Preliminary observations indicate no significant macroscopic differences among various tetracycline derivatives regarding their effects on *E. vannus*. Symptoms like slowed motility and cellular lysis appear uniform across treatments. Therefore, we hypothesize that macroscopic toxicity in non-target micro-eukaryotes is not solely determined by primary bactericidal mechanisms. Instead, it is heavily driven by the profound energetic costs of activating compensatory networks. These networks include highly differentiated transcriptional programs, detoxification processes, and antioxidant defenses. To test this hypothesis, the present study evaluates the toxicological effects of CTC, DOX, and TCH on *E. vannus*. We integrate population growth dynamics and biochemical stress markers (SOD, CAT, POD, MDA) with comparative whole-transcriptome sequencing. This approach delineates acute toxicity and long-term sublethal impacts. Furthermore, it identifies specific molecular interventions in energy flux, endoplasmic reticulum stress, and ribosomal translation. Ultimately, this research aims to reveal the intricate molecular trade-offs governing single-celled eukaryotic life under pharmaceutical stress.

## 2 Materials and methods

### 2.1 Preparation of artificial seawater

Artificial seawater was prepared using Milli-Q ultrapure water. The formulation consisted of 28.0 g of NaCl, 0.8 g of KCl, 5.0 g of MgCl₂·6H₂O, and 1.2 g of CaCl₂ dissolved per liter of water (Wang et al., **2019**; Kazmi et al., **2022**). The resulting solution was filtered through a 0.22 μm membrane. Subsequently, it was autoclaved for sterilization. Finally, the prepared seawater was stored at room temperature (Wang et al., **2025**).

### 2.2 Ciliate culture and antibiotic exposure experiments

The experimental organism, *E. vannus*, was collected from the coastline of Hongqiao Beach in Ningbo (29°46′7.95″N, 121°55′21.81″E). At the time of sampling, the water temperature was 23 °C. The salinity was 30, and the pH was 7.8. Samples were transported to the laboratory and examined under a dissecting microscope. Individual *E. vannus* specimens were isolated using a micropipette. They were then transferred into sterile culture dishes (Ji et al., **2024**). The culture medium consisted of artificial seawater filtered through a 0.22 μm membrane. This seawater was supplemented with heat-inactivated *Escherichia coli* as a food source (Wang et al., **2019**). Progeny from this initial scale-up culture were used for all subsequent experiments. To prepare the food source, a single *E. coli* colony was inoculated into Luria-Bertani (LB) broth. The culture was incubated in a shaker at 300 rpm and 37 °C for 12 h. Cells were harvested by centrifugation at 8000 rpm for 10 min. The supernatant was discarded. The *E. coli* pellet was resuspended in artificial seawater. The bacterial suspension was adjusted to an optical density (OD₆₀₀) of 1.000 (±0.001) using a UV–Vis spectrophotometer. Under sterile conditions, the suspension was transferred to 50-mL centrifuge tubes. It was incubated in a water bath at 60 °C for 2 h to inactivate the bacteria. This heat-inactivated suspension was subsequently used to cultivate *E. vannus* (Ji et al., **2024**; Wang et al., **2019**).

Preliminary experiments established the concentration ranges for acute toxicity testing. Test concentrations of 0, 2, 4, 6, 8, and 10 mg/L were prepared for CTC, DOX, and TCH using artificial seawater. Thirty *E. vannus* individuals were introduced into each well of a 12-well plate. Each well contained the respective antibiotic solution. The plates were then incubated at 25 °C for 24 h. Finally, the surviving organisms in each well were counted. Individuals exhibiting immobility or severe deformation were classified as dead.

### 2.3 Determination of growth inhibition rate and antioxidant enzyme activity

Based on preliminary results, antibiotic solutions of 0.01, 0.1, and 1 mg/L were prepared in artificial seawater. Antibiotic-free artificial seawater served as the control. The *E. vannus* suspension was exposed to these solutions in T75 cell culture flasks. The initial ciliate concentration in each flask was adjusted to 100 ind./mL. The total working volume was maintained at 100 mL (Ji et al., **2024**). Each flask received 2 mL of the inactivated *E. coli* suspension daily as a food source. The exact time of feeding was recorded. Following feeding, a 100-μL aliquot of the ciliate suspension was sampled and fixed. The cells were then counted under a microscope. This counting procedure was repeated 10 times per sample (Wang et al., **2019**). The cultures were incubated in the dark at 25 °C for a total of 144 h. Food replenishment and cell counting occurred every 24 h (Ji et al., **2024**; Wang et al., **2019**).

Samples for biochemical assays were collected under two specific exposure conditions. Acute exposure lasted for 6 h at the respective 24-h EC₅₀ concentration for each antibiotic. Chronic exposure lasted for 144 h at a sublethal concentration of 1 mg/L (Ji et al., **2024**). The malondialdehyde (MDA) content and the activities of superoxide dismutase (SOD), catalase (CAT), and peroxidase (POD) were measured. Commercial biochemical assay kits (catalog numbers BC0025, BC0170, BC0200, and BC0090, respectively) were used for these analyses. All kits were purchased from Solarbio Science & Technology Co., Ltd. (Beijing, China). The assays were performed in strict accordance with the manufacturer’s instructions (Schultz, **1997**).

### 2.4 RNA extraction, library construction, and sequencing

Total RNA was extracted from *E. vannus* samples using TRIzol reagent (Thermo Fisher, 15,596,018). The extraction followed the manufacturer’s standard operating procedures (Chomczynski and Sacchi, **1987**; Ji et al., **2024**). RNA concentration and integrity were assessed using a NanoDrop ND-1000 spectrophotometer and a Bioanalyzer 2,100 platform. High-quality RNA samples were selected for subsequent library construction. These samples required an RNA integrity number (RIN) greater than 7.0 and a total yield exceeding 1 μg.

Poly(A)-tailed mRNA was enriched from the total RNA using oligo(dT)-conjugated magnetic beads. This enrichment included two rounds of purification. The enriched mRNA was then fragmented using a magnesium-ion buffer at 94 °C. Fragmented mRNA served as a template for first-strand cDNA synthesis using reverse transcriptase. Immediately thereafter, second-strand cDNA was synthesized using *E. coli* DNA polymerase I and RNase H. A dUTP solution was incorporated into this reaction (Parkhomchuk et al., **2009**).

The synthesized blunt-ended double-stranded cDNA underwent 3′-end adenylation. Subsequently, sequencing adapters containing dual-end indices were ligated to the fragments. Uracil-DNA glycosylase (UDG) digested the dUTP-containing second strand. The remaining constructs were then enriched via PCR amplification. This specific digestion prevents amplification of the second strand. Consequently, the procedure perfectly preserves the strand-specific orientation of the transcripts. The resulting strand-specific libraries exhibited fragment sizes of 300 ± 50 bp (Parkhomchuk et al., **2009**). Following quality control, libraries were sequenced on an Illumina NovaSeq™ 6,000 platform using a paired-end 150 bp (PE150) strategy.

Raw sequencing reads were initially evaluated using FastQC. Trimmomatic then removed adapter sequences and low-quality reads. This step generated high-quality clean reads (Bolger et al., **2014**; Chen et al., **2014**). The clean reads were aligned to the *E. vannus* reference genome using HISAT2 (Kim et al., **2015**). Ciliate genomes exhibit inherent complexity. Therefore, StringTie was utilized for transcript re-assembly and quantification. This approach facilitated the identification of novel transcripts. These novel transcripts received identifiers prefixed with “MSTRG” (Pertea et al., **2015**). FeatureCounts calculated the expression levels for each gene. These expression levels were normalized as Transcripts Per Million (TPM; Liao et al., **2014**).

Principal Component Analysis (PCA) and Pearson correlation analyses were performed. These methods assessed global transcriptome variation and evaluated the reliability of biological replicates.

The biological functions of differentially expressed genes (DEGs) were investigated. Specifically, the clusterProfiler package in R performed an Over-representation Analysis (ORA). This analysis evaluated Gene Ontology (GO) terms and Kyoto Encyclopedia of Genes and Genomes (KEGG) pathways (Yu et al., **2012**). The significance threshold was established at a *q*-value < 0.05.

## 3 Results

### 3.1 Macroscopic toxicity and population growth inhibition

**Figure 1:**
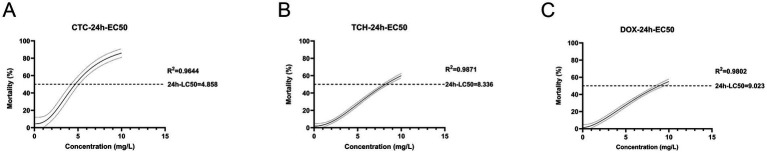
24 h-EC50 of TCs. **(A)** 24 h-EC50 of CTC; **(B)** 24 h-EC50 of TCH; **(C)** 24 h-EC50 of DOX.

This study established the acute toxicological effects of CTC, DOX, and TCH on *E. vannus*. The 24-h dose–response fitting curves revealed a distinct toxicity gradient based on EC₅₀ values. CTC exhibited the highest acute toxicity at 4.858 mg/L (Figure 1A). TCH and DOX followed with EC₅₀ values of 8.336 mg/L (Figure 1B) and 9.023 mg/L (Figure 1C), respectively.

**Figure 2:**
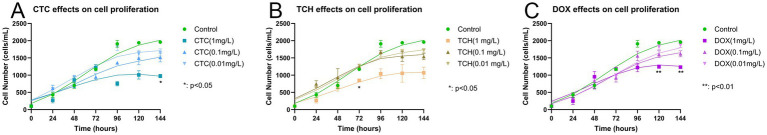
Effects of 0.01, 0.1, and 1 mg/L TCs on the growth of *Euplotes vannus* for 144 h. **(A)** The effects of CTC, 0.01, 0.1, and 1 mg/L on the growth of *E. vannus* for 144 h; **(B)** the effects of TCH, 0.01, 0.1, and 1 mg/L on the growth of *E. vannus* for 144 h; **(C)** the effects of DOX 0.01, 0.1, and 1 mg/L on the growth of *E. vannus* for 144 h; *p < 0.05,**p < 0.01.

Furthermore, a 144-h monitoring period evaluated sub-lethal concentrations (0.01, 0.1, and 1 mg/L). All three tetracyclines induced a dose-dependent inhibition of population proliferation. Exposure to 1 mg/L reduced the plateau-phase density to approximately 1,000–1,200 ind./mL (Figures 2A–C). Significant reductions in population density occurred primarily at the 1 mg/L concentration across different exposure times. Specifically, CTC induced significant differences after 144 h. TCH caused significant effects earlier at 72 h. Meanwhile, DOX elicited significant responses at both 120 h and 144 h. Based on these observations, a concentration of 1 mg/L was selected for all subsequent long-term physiological and biochemical analyses.

### 3.2 Dynamics of oxidative damage and biochemical defense

**Figure 3:**
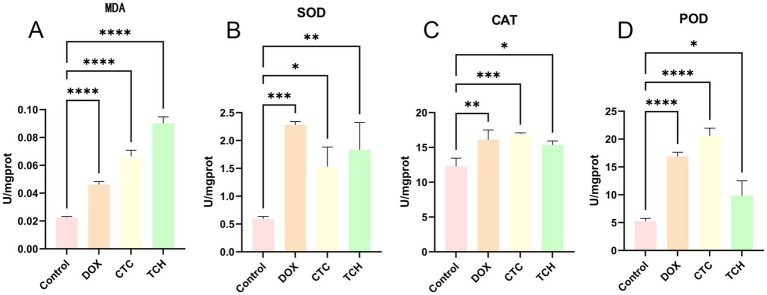
Effects of three tetracycline antibiotics on the population growth dynamics of *E. vannus* after 144 h of exposure at 1 mg/L. *p < 0.05, **p < 0.01, *** < *P*0.001, ***p < 0.001, ****p < 0.0001. **(A)** MDA (Malondialdehyde). **(B)** SOD (Superoxide Dismutase). **(C)** CAT (Catalase). **(D)** POD (Peroxidase).

**Figure 4:**
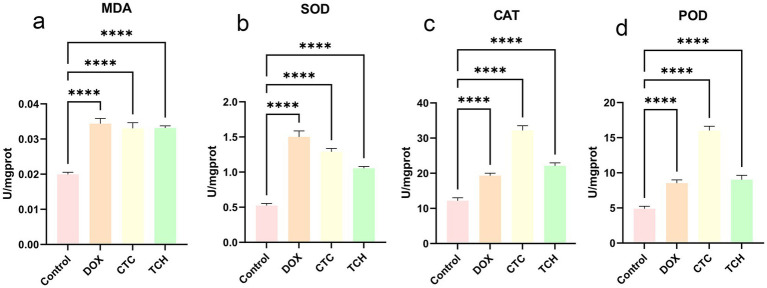
Effects of three tetracycline antibiotics on the population growth dynamics of *E. vannus* after 6 h of exposure at 24 h-EC50. *p < 0.05, **p < 0.01, *** < *P*0.001, ***p < 0.001, ****p < 0.0001. **(a)** MDA (Malondialdehyde). **(b)** SOD (Superoxide Dismutase). **(c)** CAT (Catalase). **(d)** POD (Peroxidase).

Biochemical parameters were assessed under chronic and acute exposure conditions. Chronic exposure lasted for 144 h at a concentration of 1 mg/L (Figure 3). Acute exposure lasted for 6 h at the respective 24-h EC₅₀ concentrations (Figure 4). Following chronic exposure, severe lipid peroxidation was evident across all treatments. Specifically, the TCH group accumulated the highest levels of malondialdehyde (MDA; Figure 3A). Furthermore, DOX significantly induced SOD activity (Figure 3B). Conversely, CTC elicited the most robust responses in CAT and POD activities (Figures 3C,D). Under acute exposure conditions, all three antibiotics significantly increased MDA concentrations (Figure 4a). Similarly, DOX treatment resulted in the highest SOD activity (Figure 4b). Finally, CTC treatment produced the greatest increases in CAT and POD activities (Figures 4c,d). These acute responses aligned consistently with the observations from the chronic exposure.

### 3.3 Transcriptome sequencing and expression profiles

**Figure 5:**
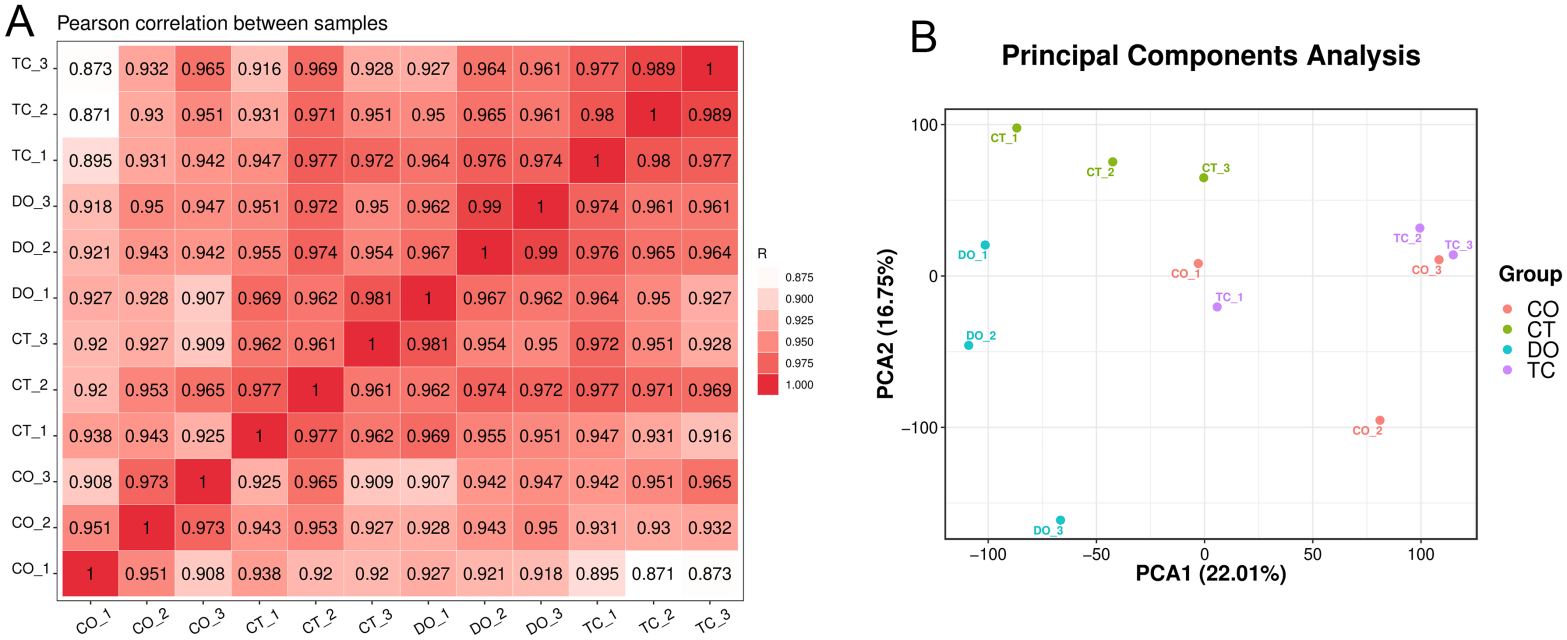
Sample correlation analysis. **(A)** Pearson correlation analysis. **(B)** PCA analysis.

RNA-seq analysis was performed on *E. vannus* exposed to the three antibiotics. This exposure lasted for 6 h at the respective 24-h EC₅₀ concentrations. Sequencing of 12 libraries generated 74.22 GB of high-quality data. The Q20 and Q30 base proportions exceeded 99.9 and 99.3%, respectively (Table 1). Pearson correlation analysis confirmed high intra-group reproducibility (R = 0.871–1.000). Principal Component Analysis (PCA) revealed clear spatial separation between the antibiotic-treated groups and the control. Specifically, DOX samples clustered tightly along the negative axis of PC1. In contrast, CTC and TCH samples formed distinct, independent clusters (Figures 5A,B).

**Table 1:** Overview of sequencing data.

Sample	RawData		ValidData		ValidRatio(reads)	Q20%	Q30%	GCcontent%
	Base	Read	Read	Base				
CO_1	48,710,642	7.31G	47,686,202	7.15G	97.90	100.00	99.43	41
CO_2	37,691,156	5.65G	36,762,338	5.51G	97.54	100.00	99.38	41
CO_3	49,853,066	7.48G	48,493,064	7.27G	97.27	100.00	99.41	40.50
CT_1	34,313,896	5.15G	33,550,186	5.03G	97.77	100.00	99.38	41
CT_2	35,697,460	5.35G	34,948,804	5.24G	97.90	100.00	99.43	41
CT_3	51,215,370	7.68G	49,323,280	7.40G	96.31	99.99	99.42	40
DO_1	34,466,288	5.17G	33,533,116	5.03G	97.29	100.00	99.31	41
DO_2	44,089,712	6.61G	43,056,400	6.46G	97.66	100.00	99.44	41
DO_3	39,830,098	5.97G	38,821,826	5.82G	97.47	100.00	99.38	41
TC_1	36,010,016	5.40G	35,067,200	5.26G	97.38	100.00	99.40	41
TC_2	45,745,196	6.86G	44,326,248	6.65G	96.90	100.00	99.38	41
TC_3	51,662,714	7.75G	49,334,084	7.40G	95.49	99.99	99.44	40

CO, Control; CT, CTC; DO, DOX; TC, TCH. Q20%, Proportion of bases with a quality score ≥ 20 (sequencing error rate < 0.01). Q30%, Proportion of bases with a quality score ≥ 30 (sequencing error rate < 0.001).

**Figure 6:**
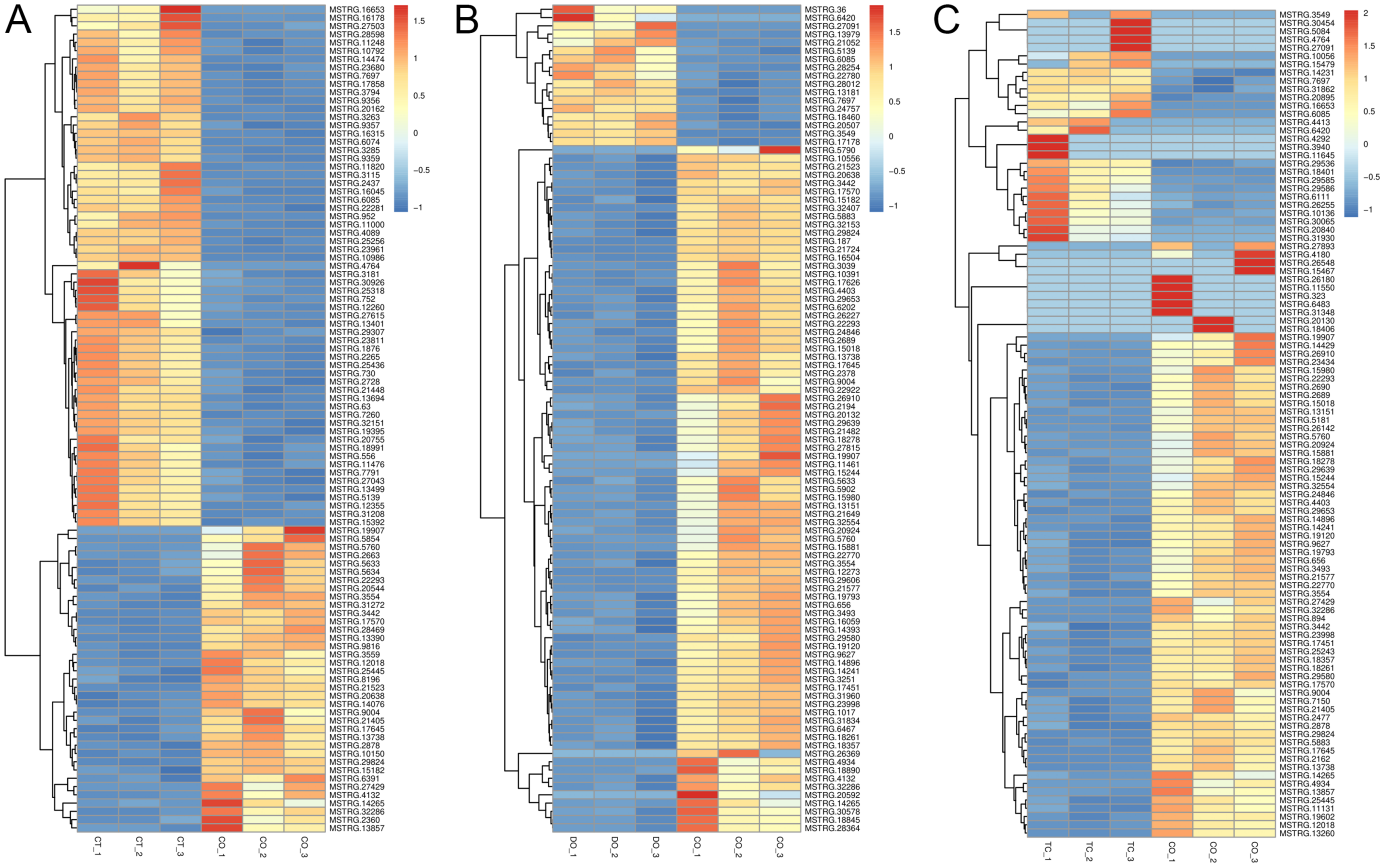
Heatmap of the Top 100 differentially expressed genes. **(A)** CTC treatment group; **(B)** DOX treatment group; **(C)** TCH treatment group. CO means Control; CT means CTC; DO means DOX; TC means TCH.

Hierarchical clustering of the top 100 most significant differentially expressed genes (DEGs) highlighted profound transcriptional reprogramming. In the CTC group, 84% of the core DEGs exhibited pronounced downregulation (Figure 6A). This pattern indicates a state of global transcriptional repression. The DOX group demonstrated acute repression of routine metabolic genes. Simultaneously, it exhibited rapid activation of an inducible defense cluster (Figure 6B). Similarly, the TCH group displayed massive repression of housekeeping genes. This repression coincided with a sharp upregulation of acute stress-induced gene networks (Figure 6C).

### 3.4 Functional enrichment analysis of DEGs

#### 3.4.1 GO/KEGG enrichment of CTC

**Figure 7:**
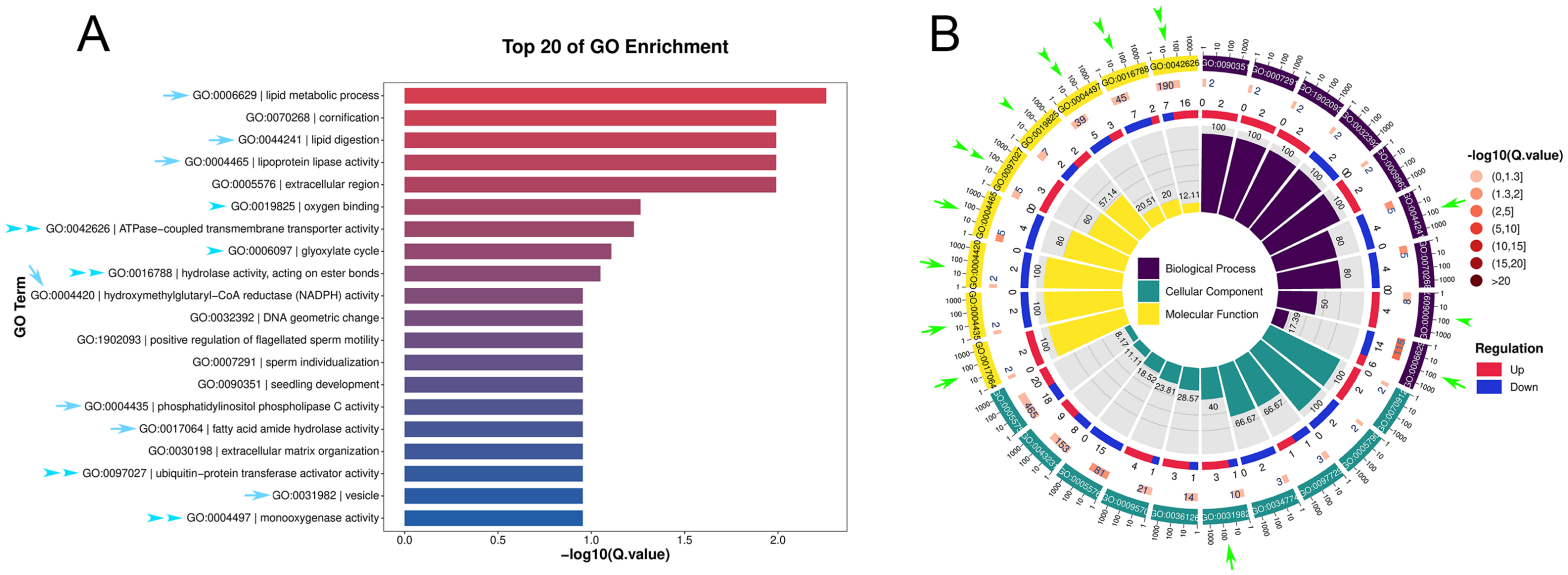
GO enrichment analysis of the CTC group. **(A)** TOP 20 of GO enrichment. Blue arrow point to lipid metabolism and cell membrane remodeling terms, blue arrowhead point to energy metabolism terms, blue double arrowhead point to detoxication terms. **(B)** The results of the GO enrichment analysis are presented in the form of a LoopCircos. Green arrow point to lipid metabolism and cell membrane remodeling terms, green arrowhead point to energy metabolism terms, green double arrowhead point to detoxication terms.

Gene Ontology (GO) enrichment analysis identified seven terms related to lipid metabolism and cell membrane remodeling. These specific terms included lipid metabolic process (GO: 0006629), lipid digestion (GO: 0044241), and lipoprotein lipase activity (GO: 0004465). Additional terms were hydroxymethylglutaryl-CoA reductase (NADPH) activity (GO: 0004420), phosphatidylinositol phospholipase C activity (GO: 0004435), fatty acid amide hydrolase activity (GO: 0017064), and vesicle (GO: 0031982). Furthermore, the analysis highlighted two terms associated with energy metabolism. These were oxygen binding (GO: 0019825) and the glyoxylate cycle (GO: 0006097). Four detoxification-related terms were also significantly enriched. These comprised ATPase-coupled transmembrane transporter activity (GO: 0042626), hydrolase activity acting on ester bonds (GO: 0016788), ubiquitin-protein transferase activator activity (GO: 0097027), and monooxygenase activity (GO: 0004497; Figure 7A). Notably, nine of the top 20 enriched GO terms belonged to the molecular function category (Figure 7B).

**Figure 8:**
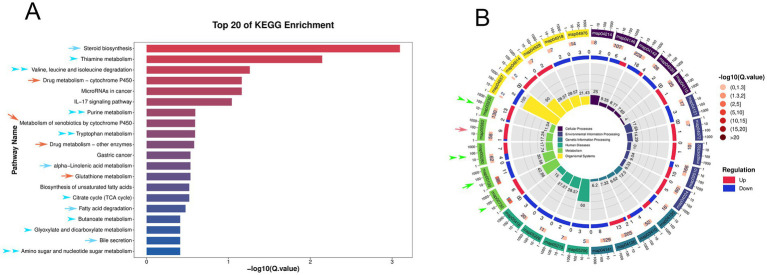
KEGG enrichment analysis of the CTC group. **(A)** TOP 20 of KEGG enrichment. Blue arrow point to KEGG pathway related to lipid metabolism and cell membrane remodeling, blue arrowhead point to KEGG pathway related to energy metabolism and core carbon metabolism, blue double arrowhead point to KEGG pathway related to amino acids and alterations in basic metabolism, orange arrow showed that KEGG pathway of drug metabolism and the antioxidant detoxification system. **(B)** The results of the KEGG enrichment analysis are presented in the form of a LoopCircos. Green arrow point to lipid metabolism and cell membrane remodeling pathway, green arrowhead point to energy metabolism pathway, green double arrowhead point to amino acids and alterations in basic metabolism, pink arrow showed that KEGG pathway of detoxication.

Kyoto Encyclopedia of Genes and Genomes (KEGG) pathway enrichment analysis identified the top 20 altered pathways. Four pathways were associated with lipid metabolism and cell membrane remodeling. Another four pathways related to the regulation of energy and core carbon metabolism. Additionally, four pathways were linked to amino acid and basic metabolic alterations. Finally, four pathways involved drug metabolism and the antioxidant detoxification system (Figure 8A). The cytochrome P450 drug metabolism pathway (map00982) was significantly upregulated. This specific pathway comprised nine upregulated genes and one downregulated gene. Conversely, 11 genes in the steroid biosynthesis pathway (map00100) were significantly downregulated. Six genes in the thiamine metabolism pathway (map00730) also exhibited significant downregulation. Within the purine metabolism pathway (map00280), seven genes were upregulated and four were downregulated. Furthermore, the valine, leucine, and isoleucine degradation pathway (map00280) contained 13 upregulated genes and two downregulated genes (Figure 8B).

#### 3.4.2 GO/KEGG enrichment of DOX

**Figure 9:**
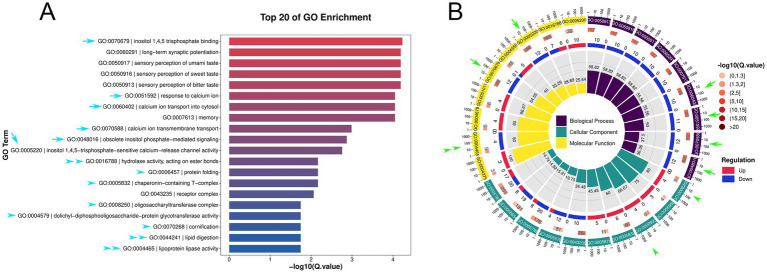
GO enrichment analysis of the DOX group. **(A)** TOP 20 of GO enrichment. Blue arrow point to Ca^2+^ and IP3 Cascade terms, blue arrowhead point to protein homeostasis and organelle stress terms, blue double arrowhead point to lipid metabolism terms. **(B)** The results of the GO enrichment analysis are presented in the form of a LoopCircos. Green arrow point to Ca^2+^ and IP3 Cascade terms, green arrowhead point to protein homeostasis and organelle stress terms, green double arrowhead point to lipid metabolism terms.

GO enrichment analysis identified six terms related to the Ca^2+^ and IP₃ cascade. These specific terms included inositol 1,4,5-trisphosphate binding (GO:0070679), response to calcium ion (GO: 0051592), and calcium ion transport into the cytosol (GO: 0060402). Additional terms in this category were calcium ion transmembrane transport (GO: 0070588), obsolete inositol phosphate-mediated signaling (GO: 0048016), and inositol 1,4,5-trisphosphate-sensitive calcium-release channel activity (GO: 0005220). Furthermore, the analysis highlighted five terms associated with protein homeostasis and organelle stress. These comprised protein folding (GO: 0006457), chaperonin-containing T-complex (GO: 0005832), and oligosaccharyltransferase complex (GO: 0008250). Other terms in this group were dolichyl-diphosphooligosaccharide-protein glycotransferase activity (GO: 0004579) and cornification (GO: 0070268). Additionally, three terms were related to lipid metabolism. These included hydrolase activity acting on ester bonds (GO: 0016788), lipid digestion (GO: 0044241), and lipoprotein lipase activity (GO: 0004465; Figure 9A). Among the top 20 enriched terms, most genes were significantly downregulated. However, the terms GO: 0008250 and GO: 0005832 were significantly upregulated (Figure 9B).

**Figure 10:**
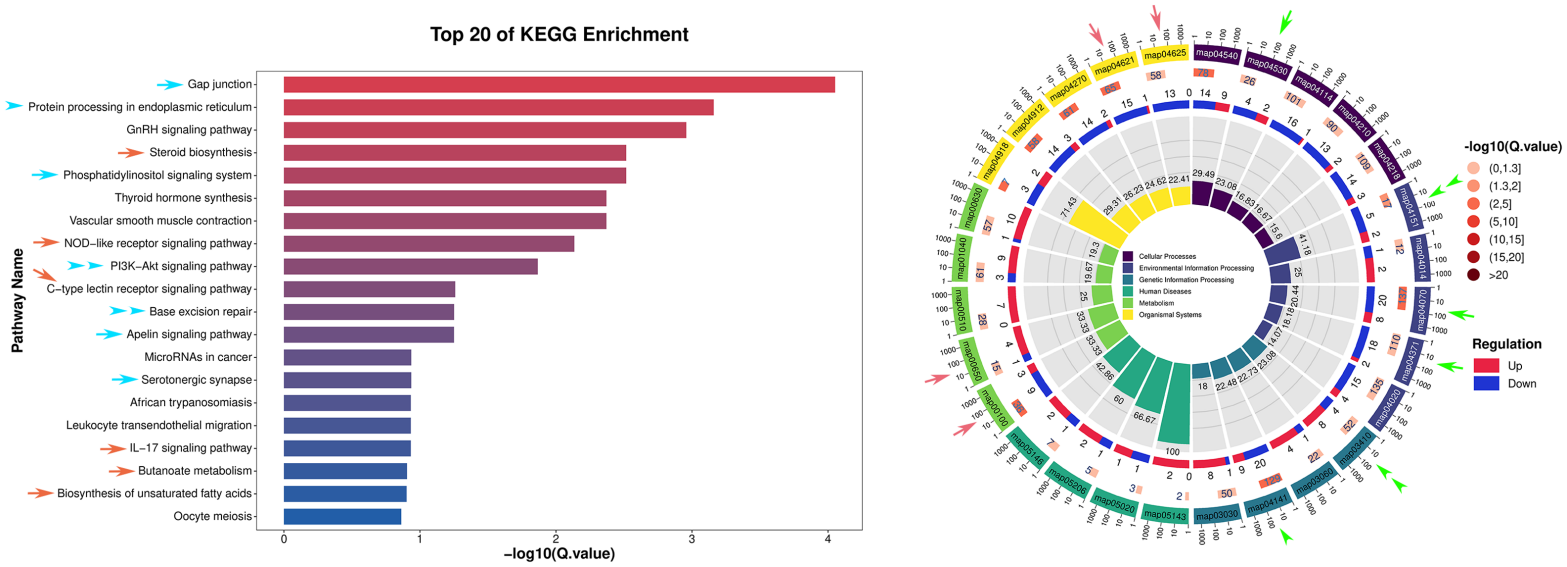
KEGG enrichment analysis of the DOX group. **(A)** TOP 20 pathway of KEGG enrichment. Blue arrow point to KEGG pathway related to IP3-Calcium Signaling, blue arrowhead point to KEGG pathway related to endoplasmic reticulum stress and protein quality control, blue double arrowhead point to KEGG pathway related to amino acids and alterations in DNA repair-related pathway, orange arrow showed that KEGG pathway of membrane system remodeling and energy metabolism-related. **(B)** The results of the KEGG enrichment analysis are presented in the form of a LoopCircos. Green arrow point to IP3-Calcium Signaling pathway, green arrowhead point to endoplasmic reticulum stress and protein quality control pathway, green double arrowhead point to amino acids and alterations in DNA repair-related, pink arrow showed that KEGG pathway of membrane system remodeling and energy metabolism-related.

KEGG pathway enrichment analysis identified the top 20 altered pathways. Four pathways were associated with the Ca^2+^ and IP₃ cascade. One pathway related to endoplasmic reticulum stress. Two pathways were linked to DNA repair. Additionally, six pathways involved regulating lipid and steroid metabolism, as well as remodeling cellular membrane defense (Figure 10A). Three key communication and sensing pathways centered around Ca^2+^ and IP₃ exhibited significant downregulation and inhibition. These specific pathways included the gap junction, phosphatidylinositol signaling system, and apelin signaling pathways. The protein processing in endoplasmic reticulum pathway contained nine upregulated genes and 20 downregulated genes. The PI3K-Akt signaling pathway (map04151) was significantly downregulated. Conversely, the base excision repair pathway (map04151) was upregulated. The NOD-like receptor signaling pathway (map04621) and the C-type lectin receptor signaling pathway (map04625) were both downregulated. The steroid biosynthesis pathway (map00100) also exhibited significant downregulation. In contrast, the butanoate metabolism pathway (map00650) was upregulated (Figure 10B).

#### 3.4.3 GO/KEGG enrichment of TCH

**Figure 11:**
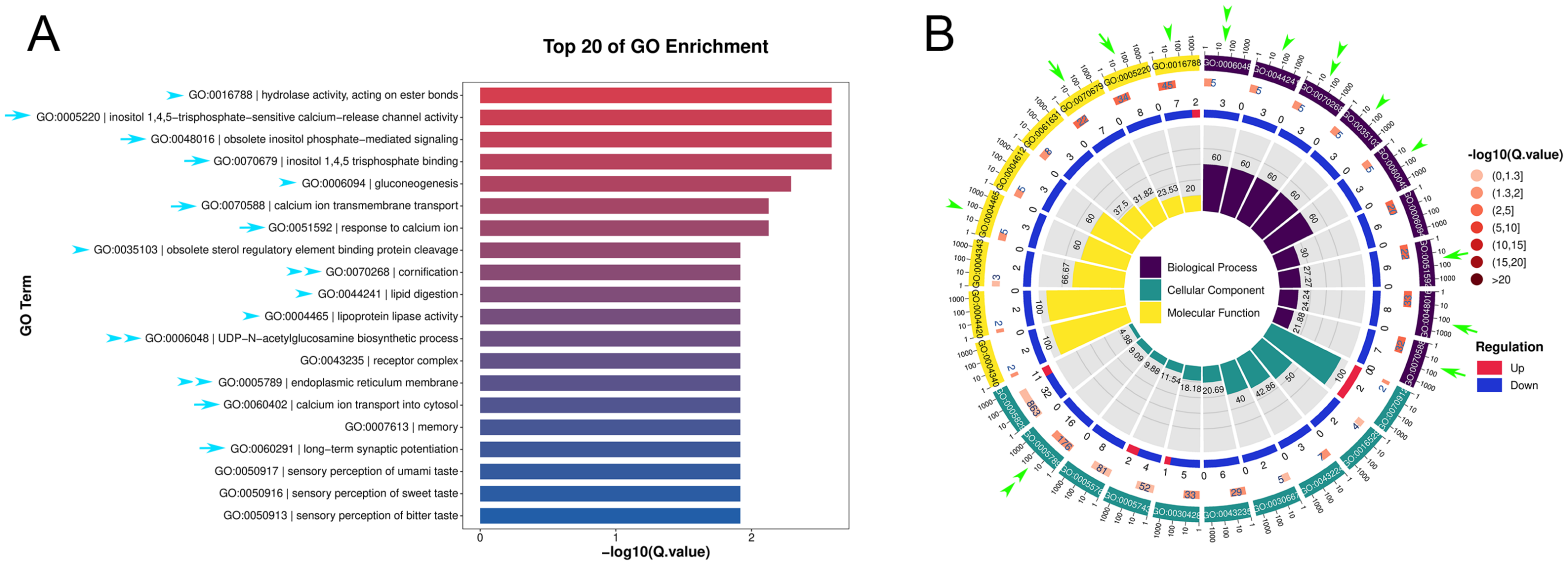
GO enrichment analysis of the TCH group. **(A)** TOP 20 of GO enrichment. Blue arrow point to Ca^2+^ and IP3 Cascade terms, blue arrowhead point to energy provision and lipid remodeling terms, blue double arrowhead point to cell structure and defense terms. **(B)** The results of the GO enrichment analysis are presented in the form of a LoopCircos. Green arrow point to Ca^2+^ and IP3 Cascade terms, green arrowhead point to energy provision and lipid remodeling terms, green double arrowhead point to cell structure and defense terms.

GO enrichment analysis identified seven terms related to the Ca^2+^ and IP₃ cascade. Additionally, five terms were associated with lipid remodeling and energy provision. Three terms were linked to cell structure and defense (Figure 11A). Five terms within the Ca^2+^ and IP₃ cascade were significantly downregulated. Furthermore, all five terms related to lipid remodeling and energy provision exhibited significant downregulation. Similarly, all three terms associated with cell structure and defense were significantly downregulated (Figure 11B).

**Figure 12:**
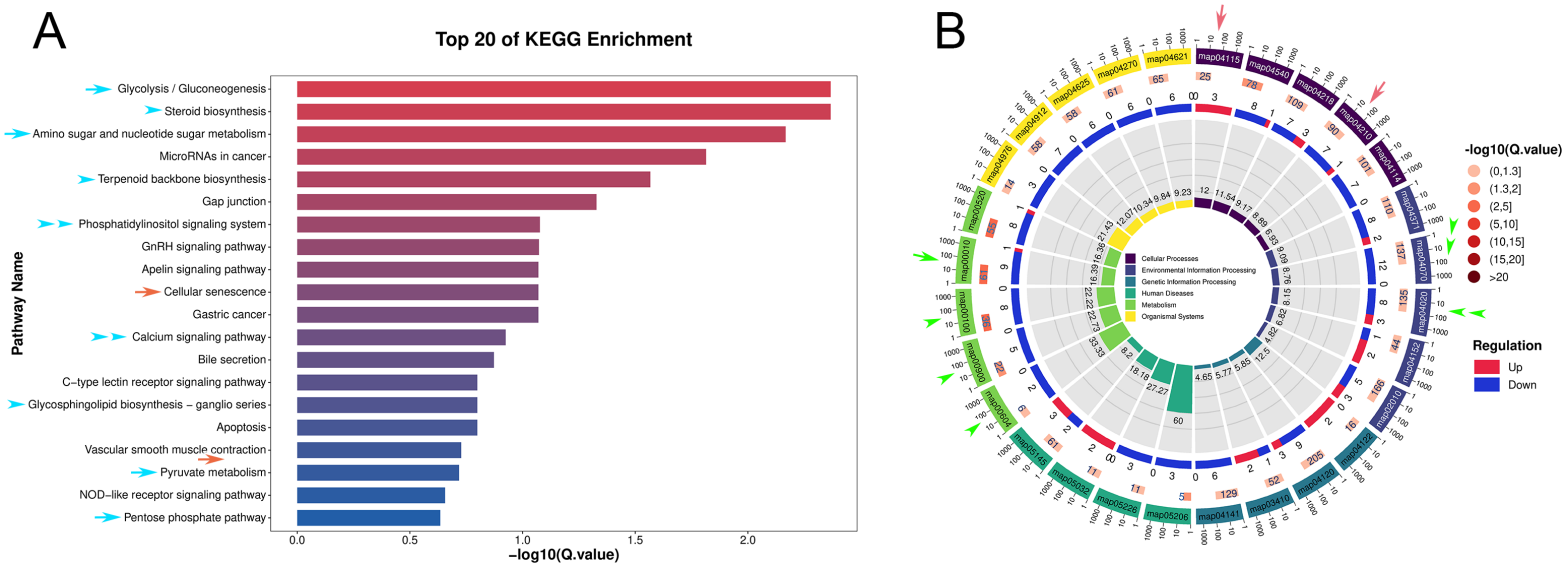
KEGG enrichment analysis of the TCH group. **(A)** TOP 20 pathway of KEGG enrichment. Blue arrow point to KEGG pathway related to energy metabolism and antioxidant, blue arrowhead point to KEGG pathway related to cell membrane structural repair, blue double arrowhead point to KEGG pathway related to IP3-Calcium Signaling pathway, orange arrow showed that KEGG pathway of cellular senescence and apoptosis. **(B)** The results of the KEGG enrichment analysis are presented in the form of a LoopCircos. Green arrow point to KEGG pathway related to energy metabolism and antioxidant, green arrowhead point to KEGG pathway related to cell membrane structural repair, green double arrowhead point to KEGG pathway related to IP3-Calcium Signaling pathway, pink arrow showed that KEGG pathway of cellular senescence and apoptosis.

KEGG pathway enrichment analysis identified several key alterations. Four pathways were associated with the remodeling of energy metabolism and antioxidant defense. Three pathways related to lipid and sterol metabolism. Two pathways were linked to the Ca^2+^ and IP₃ cascade. Additionally, two pathways involved cellular senescence and apoptosis (Figure 12A). The glycolysis and gluconeogenesis pathways underwent significant regulation and inhibition. The steroid biosynthesis (map00100), terpenoid backbone biosynthesis (map00900), and ganglio series glycosphingolipid biosynthesis (map00604) pathways were all significantly downregulated. Two pathways associated with the Ca^2+^ and IP₃ cascade also exhibited significant downregulation. Conversely, the cellular senescence pathway (map04115) was significantly upregulated. Finally, the apoptosis pathway (map04210) was slightly downregulated.

## 4 Discussion

While all three conditions share some common stress responses (like the suppression of steroid biosynthesis), their primary mechanisms of action and the resulting cellular fates are completely different.

### 4.1 CTC 24 h-EC50, 6 h to *Euplotes vannus*: active detoxification and metabolic reprogramming

Exposure to CTC triggered a robust defense mechanism in *E. vannus*. The organism detoxified the xenobiotic while simultaneously shifting its energy sources to sustain a high-energy defensive state. A massive detoxification response was evident. Specifically, the cytochrome P450 pathway (map00982; Lv et al., **2021**; Zhang et al., **2025**), monooxygenase activity, and various transmembrane transporters were significantly upregulated. These molecular changes indicate that *E. vannus* actively metabolized and exported CTC. Furthermore, the organism reprogrammed its metabolism to secure alternative energy sources. The valine, leucine, and isoleucine degradation pathway was upregulated. This indicates the catabolism of branched-chain amino acids to fuel the tricarboxylic acid (TCA) cycle. Such alternative energy sourcing likely compensated for stress on primary energy pathways (Pei et al., **2021**). Finally, the results highlighted extensive membrane remodeling. Lipid metabolic processes, including lipoprotein lipase, fatty acid amide hydrolase (FAAH), and phosphatidylinositol phospholipase C (PIPC) activities, were highly active. Concurrently, the steroid biosynthesis pathway was downregulated. These alterations suggest that *E. vannus* modified its membrane composition. This remodeling likely restricted drug influx or facilitated the formation of vesicles (GO:0031982) for drug efflux (Shu et al., **2025**).

### 4.2 DOX 24 h-EC50, 6 h to *Euplotes vannus*-triggered endoplasmic reticulum stress and signal transduction overload

DOX caused severe intracellular damage. This damage specifically targeted DNA and protein folding machinery. Consequently, normal cellular signaling collapsed, and survival pathways shut down. Furthermore, the upregulation of the base excision repair pathway (map04151) confirmed direct DOX-induced DNA damage (Yarmohammadi et al., **2021**). This damage triggered severe endoplasmic reticulum stress. Chaperonin complexes (GO:0005832) and oligosaccharyltransferase (GO:0008250) were significantly upregulated. This upregulation indicates an acute cellular response to manage misfolded proteins. A severe collapse of Ca^2+^ and IP₃ signaling emerged as a hallmark of DOX toxicity (Renu et al., **2018**). Extensive downregulation occurred across multiple Ca^2+^ and IP₃ pathways. These included gap junctions, the phosphatidylinositol signaling system, and the apelin signaling pathway. This widespread downregulation impaired the ability of *E. vannus* to regulate intracellular calcium. Such calcium dysregulation serves as a primary trigger for organelle stress and subsequent cell death. Finally, DOX exposure suppressed key survival and immune mechanisms. The PI3K-Akt signaling pathway represents a master regulator of cellular survival and growth. Its extensive downregulation halted *E. vannus* proliferation and increased cellular vulnerability (Octavia et al., **2012**). Additionally, NOD-like and C-type lectin receptors were suppressed. This suppression indicates a critical failure in inflammatory and immune sensing mechanisms.

### 4.3 TCH 24 h-EC50, 6 h to *Euplotes vannus*-induced systemic paralysis and structural defense trade-offs

DOX caused acute intracellular damage. CTC triggered an active detoxification defense. In contrast, TCH induced severe metabolic starvation and energy collapse in *E. vannus*. TCH exposure broadly suppressed the synthesis of core structural components. Specifically, the glycolysis and gluconeogenesis pathways were significantly inhibited. Consequently, *E. vannus* failed to metabolize glucose to fulfill basic energy demands. Furthermore, the synthesis of major structural lipids was entirely shut down. These inhibited pathways included steroid, terpenoid backbone, and glycosphingolipid biosynthesis. Apoptosis is an active, energy-dependent process. It requires sufficient ATP and precise Ca^2+^ signaling. However, TCH-induced resource depletion and signaling collapse prevented this programmed cell death. As a result, the apoptosis pathway exhibited slight downregulation. Instead, *E. vannus* entered cellular senescence (map04115; Clarke et al., **2012**; Pinton et al., **2008**). The cells ceased division and arrested their cell cycle. Ultimately, profound structural and metabolic exhaustion forced the organism into a dormant, senescent state.

In conclusion, despite potentially sharing structural similarities, exposure to CTC, DOX, and TCH induces fundamentally divergent cellular fates and toxicological mechanisms in the marine ciliate *E. vannus*. While CTC triggers active detoxification and metabolic reprogramming, DOX causes profound intracellular damage and signal transduction collapse, and TCH leads to systemic metabolic starvation and cellular senescence. These distinct molecular responses highlight the high complexity and chemical-specific nature of xenobiotic interactions in marine microeukaryotes. Therefore, the findings of this study not only clarify the specific toxicological profiles of these antibiotics but also provide important reference significance for the ecological toxicity assessment and risk prediction of other emerging marine pollutants. Specifically, it underscores the necessity of utilizing mechanism-based, multi-omics evaluations rather than relying on generalized toxicity models when assessing the environmental risks of novel anthropogenic contaminants in marine ecosystems.

### 4.4 Divergent cellular fates in *Euplotes vannus*: reference significance for the Ecotoxicological assessment of emerging pollutants

In conclusion, CTC, DOX, and TCH share structural similarities. However, exposure to these antibiotics induces fundamentally divergent cellular fates and toxicological mechanisms in the marine ciliate *E. vannus*. CTC triggers active detoxification and metabolic reprogramming. DOX causes profound intracellular damage and signal transduction collapse. Meanwhile, TCH leads to systemic metabolic starvation and cellular senescence. These distinct molecular responses highlight the high complexity of xenobiotic interactions in marine microeukaryotes. They also emphasize the chemical-specific nature of these interactions. Therefore, the findings of this study clarify the specific toxicological profiles of these antibiotics. Furthermore, they serve as a critical reference for the ecological toxicity assessment and risk prediction of other emerging marine pollutants. Ultimately, these results underscore the necessity of mechanism-based, multi-omics evaluations. Researchers must utilize these comprehensive approaches rather than relying on generalized toxicity models to assess the environmental risks of novel anthropogenic contaminants in marine ecosystems.

## Data Availability

The datasets presented in this study can be found in online repositories. The names of the repository/repositories and accession number(s) can be found in the article/supplementary material.
